# Treatment strategies for erosive genital lichen planus: A systematic review of therapeutic modalities and emerging breakthroughs

**DOI:** 10.1002/hsr2.70129

**Published:** 2024-10-14

**Authors:** Borna Safari‐Kish, Matin Bidares, Shirin Zaresharifi, Hesam Malekzadeh‐Shoushtari, Mahsa Aziz, Mahsa Salehi, Khatere Zahedi

**Affiliations:** ^1^ Clinical Research Development Center, Najafabad Branch Islamic Azad University Najafabad Iran; ^2^ Skin Research Center Shahid Beheshti University of Medical Sciences Tehran Iran; ^3^ School of Medicine Ahvaz Jundishapur University of Medical Sciences Ahvaz Iran; ^4^ Department of Pathology, School of Medicine Mazandaran University of Medical Sciences Mazandaran Iran

**Keywords:** Erosive genital lichen planus, EGLP, lichen planus, treatment

## Abstract

**Background and Aims:**

Erosive genital lichen planus (EGLP) is a severe form of lichen planus characterized by painful erosions in the genital area, leading to significant distress and complications. This review aims to provide a thorough examination of EGLP, focusing on its clinical manifestations, diagnostic challenges, and various treatment strategies, with an emphasis on patient‐centered care.

**Methods:**

Adhering to PRISMA guidelines, we conducted a systematic review of 26 studies that explored dermatological treatments for EGLP. Our literature search was comprehensive, covering PubMed, Scopus, and Web of Science. Data were extracted systematically using established tools to ensure a robust analysis of the treatment modalities.

**Results:**

EGLP presents with a range of symptoms, including severe pain, itching, and sexual dysfunction. Treatment options include topical corticosteroids, systemic medications, and surgical interventions. Despite the availability of various therapies, many cases are refractory to treatment, resulting in chronic symptoms and reduced quality of life. Emerging therapies show promise but are not yet established as standard practice.

**Conclusion:**

Management of EGLP requires a tailored, multidimensional approach. While topical corticosteroids remain essential, the development of new therapies offers hope for improved outcomes. A patient‐centered approach is vital to address both the physical and psychosocial impacts of EGLP. Continued research is necessary to refine treatment protocols and enhance patient care.

## 1 INTRODUCTION

Erosive genital lichen planus (EGLP) stands as a distinctive subtype within the spectrum of lichen planus (LP), marked by erosions affecting the vulva, vaginal introitus, and penis, resulting in scarring, discomfort, and complications in genitourinary and sexual function, particularly in postmenopausal women, necessitating a multidimensional approach to its management.^1^, ^2^, ^3^

The autoimmune nature of LP, drawing attention to the T‐cell‐mediated attack on basal keratinocytes and the characteristic histological features observed in affected tissues.^4^

This condition, while more commonly observed in women, also manifests in men, often presenting with an annular configuration on the glans penis.^5^ The incidence of isolated cases remains elusive, making EGLP a challenging condition to quantify accurately. As the narrative unfolds, the focus broadens to LP affecting various mucous membranes, emphasizing its immunologically mediated inflammatory nature.^6^

The prevalence of vulvovaginal LP, though not precisely defined, is explored alongside its diverse clinical presentations. The association with autoimmune diseases, especially thyroid disease and vitiligo, is highlighted, emphasizing the need for a comprehensive understanding of LP's systemic implications.^7^, ^8^ Despite the diverse manifestations of LP, the article establishes a unifying thread through the commonality of inflammatory autoimmune origins.^6^ The challenge of differential diagnosis, especially in the context of EGLP, prompts an exploration of treatment strategies. The significance of longitudinal data and patient‐reported outcomes emerges as a critical aspect, urging a holistic approach to managing LP, taking into account the impact on patients’ quality of life.

In the realm of LP management, corticosteroids of moderate to high potency have been a conventional approach, demonstrating efficacy in controlling symptoms of LP that are popular or plaque‐type. However, the treatment landscape for erosive genital disease, such as EGLP, proves to be more intricate.^9^ Although limited in size and scope, a collection of individual cases and a controlled study with a placebo group have supported topical corticosteroids as first‐line therapy, a lack of overwhelming evidence complicates the determination of a singular treatment approach.^9^, ^10^, ^11^ Our exploration encompasses the clinical nuances, diagnostic challenges, and therapeutic strategies, including tacrolimus ointment, intralesional triamcinolone injections, and various immunomodulatory and immunosuppressant therapies, ranging from systemic corticosteroids to hydroxychloroquine, TNF alpha blockers, cyclosporine, azathioprine, mycophenolate mofetil, adalimumab, and methotrexate.^12^ The selection of the second‐line therapy is often influenced by anecdotal clinical expertise and an assessment of the risk‐benefit ratio associated with each medicine.

In this systematic review, the therapeutic challenges of erosive genital LP extend to EGLP, where the use of potent topical corticosteroids has been a cornerstone, despite challenges such as resistance and steroid‐related side effects. The article navigates through the intricacies of treatment choices and their implications, emphasizing the importance of individualized approaches based on clinic.

## 2 METHODS

### 2.1 Objective

**Figure 1:**
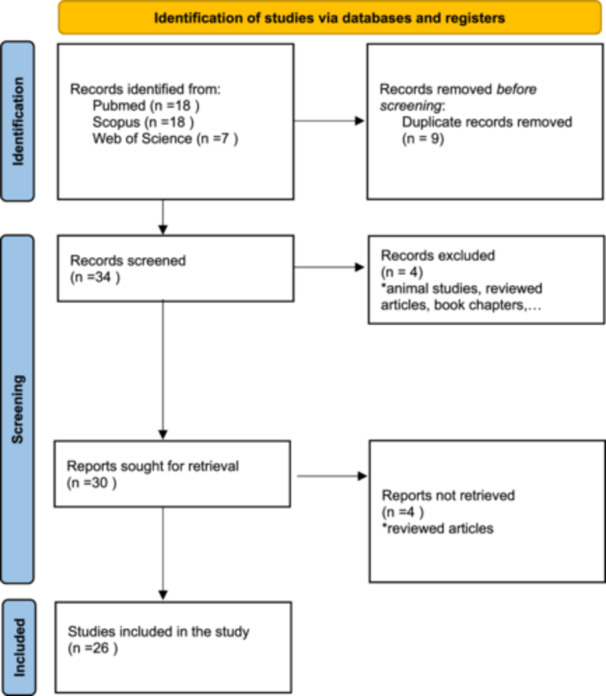
The flow chart of study selection. *From*: Page MJ, McKenzie JE, Bossuyt PM, Boutron I, Hoffmann TC, Mulrow CD, et al. The PRISMA 2020 statement: an updated guideline for reporting systematic reviews. BMJ 2021;372:n71. doi: 10.1136/bmj.n71. For more information, visit: http://www.prisma-statement.org/.

The aim of this systematic review is to assess the correlation between EGLP and various treatment modalities within the domain of dermatology. Our approach adheres to established standards, following the PRISMA guidelines (Preferred Reporting Items for Systematic Reviews and Meta‐analyses) (Figure 1).

### 2.2 Literature search

A comprehensive literature search was conducted up to [15th September 2023], retrieving relevant papers from PubMed, Scopus, and Web of Science. The search strategy comprised four primary categories of keywords and Medical Subject Headings (MeSH). (Table 1) The search methodology followed the PRISMA guidelines and involved using the Boolean operator “AND” to combine four groups of keywords, ensuring that each article included terms from all specified groups. Within each group, the Boolean operator “OR” was used to capture all relevant variations and synonyms, enhancing the comprehensiveness of our search.

**Table 1:** Search strategies.

Group	Keywords	MESH Terms	Tiab Terms
Group 1 (Lichen planus)	(Lichen planus OR LP OR Oral lichen planus OR Cutaneous lichen planus OR Mucosal lichen planus OR Lichen planus treatment OR Lichen planus symptoms OR Lichen planus diagnosis OR Lichen planus etiology OR Lichen planus pathogenesis)	“Lichen Planus”[Mesh] OR “Lichen Planus, Oral”[Mesh]	“Lichen planus follicularis” [Supplementary Concept] OR “Lichen Planus, Familial” [Supplementary Concept] OR LP[tiab] OR “Cutaneous lichen planus”[tiab] OR “Mucosal lichen planus”[tiab]
Group 2 (Genitalia)	(Genitalia OR Male genitalia OR Female genitalia)	“Genitalia”[Mesh] OR “Genitalia, Male”[Mesh] OR “Genitalia, Female”[Mesh]	
Group 3 (Erosive conditions)	(Erosive OR Erosion OR Erosive disease OR Erosive lichen planus)		Erosive[tiab] OR Erosion[tiab] OR “Erosive diseas*”[tiab] OR “Erosive lichen planus”[tiab]
Group 4 (Treatment)	(“therapy” OR “Treatment Outcome” OR “Treatment Failure” OR “Emergency Treatment” OR “Treatment Refusal” OR “Treatment Switching” OR “Conservative Treatment” OR “Neoadjuvant Therapy” OR “Clinical Protocols” OR “Behavior Therapy” OR “Duration of Therapy” OR “Therapeutics” OR “Time‐to‐Treatment” OR “Drug Therapy” OR “drug therapy” OR Treatment OR Management OR “Intervention*” OR “Medical treatment” OR Pharmacotherapy OR “Therapeutic approach*” OR “Clinical management*” OR “Treatment option*” OR “Treatment strateg*” OR “Adrenal Cortex Hormones” OR corticosteroid* OR steroids OR retinoids OR “Antibacterial Agents”)	“Treatment Outcome”[Mesh] OR “Treatment Failure”[Mesh] OR “Emergency Treatment”[Mesh] OR “Treatment Refusal”[Mesh] OR “Treatment Switching”[Mesh] OR “Conservative Treatment”[Mesh] OR “Neoadjuvant Therapy”[Mesh] OR “Clinical Protocols”[Mesh] OR “Behavior Therapy”[Mesh] OR “Duration of Therapy”[Mesh] OR “Therapeutics”[Mesh] OR “Time‐to‐Treatment”[Mesh] OR “Drug Therapy”[Mesh] OR “Adrenal Cortex Hormones”[Mesh] OR corticosteroid*[tiab] OR steroids[Mesh] OR retinoids[Mesh] OR “Immunosuppressive Agents”[Mesh] OR “Antibacterial Agents”[Mesh]	“therapy” [Subheading] OR “drug therapy” [Subheading] OR Treatment[tiab] OR Management[tiab] OR “Intervention*”[tiab] OR “Medical treatment”[tiab] OR Pharmacotherapy[tiab] OR “Therapeutic approach*”[tiab] OR “Clinical management*”[tiab] OR “Treatment option*”[tiab] OR “Treatment strateg*”[tiab] OR

The search methodology aligned with the specific query format for each database. To mitigate the risk of overlooking relevant literature, a thorough examination of the reference lists of pertinent systematic reviews was conducted, and works within the scope of our research were included. Each stage was conducted by two independent reviewers, and any discrepancies were resolved through discussions between the reviewers.

### 2.3 Data extraction and study quality assessment

Two independent reviewers evaluated the title and abstract of each study to determine eligibility for inclusion in this systematic review. Studies not meeting the inclusion criteria were excluded. The full text of the remaining studies underwent screening, and those deemed eligible entered the data extraction phase. The subsequent step involved gathering information in four categories: 1. research characteristics (authors, location, year, and research type); 2. patient‐specific variables (age and gender); 3. study design (participant count, sampling strategy, and confounding factors); and 4. outcomes (covering EGLP manifestations and treatment modalities). The Joanna Briggs Institute critical appraisal checklists for dermatological research (available at https://jbi.global/critical-appraisal-tools) were utilized by the two reviewers for cohort, case–control, and analytical cross‐sectional research. In cases of disagreement, a third author participated in the resolution process.

### 2.4 Protocol registration

This review protocol was registered with OSF, an international database for systematically reported dermatology reviews.

This structured method aligns with the format you provided for the “Methods” section of a systematic review. Adjustments can be made based on specific requirements or focus areas.

## 3 RESULTS

A total of 26 articles were reviewed, including 17 case reports, 5 original studies, 3 case series, and 1 randomized controlled trial protocol. A total of 26 articles were reviewed, with lichen planus affecting the female or male genitalia being the primary focus (Table 2). EGLP was frequently reported, while non‐erosive subtypes were less commonly described.

**Table 2:** Included articles and their information.

Article	Year	Study type	Summary	Key findings
Vaskovskaya and Mirkhodzhaeva	1974	Retrospective Analysis	Among 341 patients with buccal lichen planus, 24.6% had simultaneous genital involvement. Mainly observed in 40–60‐year‐olds with exudative hyperemic and erosive ulcerative forms.	Simultaneous involvement of buccal and genital mucous membranes. Common in 40–60 year‐olds. Hyperemic and erosive ulcerative forms.
Edwards	1989	Case Series	Investigated 7 cases of vulvar lichen planus.	Explored clinical presentation and management of vulvar lichen planus in 7 cases.
Bermejo et al.	1990	Case Series	Presented five cases with lichen planus involving oral cavity and genitalia. Ages ranged from 40 to 65 years. Four patients had associated systemic disorders.	Cases demonstrated lichen planus affecting oral and genital areas, often associated with systemic disorders.
Cribier et al.	1993	Case Report	Described a 52‐year‐old man with peno‐gingival syndrome, a male equivalent of vulvo‐vagino‐gingival syndrome. Chronic erosive gingivitis associated with eroded genital lesions.	Male equivalent of vulvo‐vagino‐gingival syndrome. Chronic erosive gingivitis and eroded genital lesions.
Porter et al.	2001	Case Report	Reported a case of erosive penile lichen planus in a 27‐year‐old uncircumcised man. Symptoms improved after circumcision.	Erosive penile lichen planus treated with circumcision.
Mous et al.	2002	Case Report	Three women aged 46, 48, and 73 years suffered from erosive genital lichen planus. Prominent signs included vulvar irritation, dyspareunia, and vaginal discharge.	Cases of erosive genital lichen planus in women with prominent signs of irritation, dyspareunia, and discharge.
Al‐Mutairi et al.	2005	Case Report	A 54‐year‐old male with acute generalized lichen planus treated with weekly betamethasone 5‐mg oral mini‐pulse therapy.	Successful treatment of acute generalized lichen planus with oral mini‐pulse therapy.
Lonsdale‐Eccles and Velangi	2005	Case Series	Eleven patients with erosive lichen planus treated with topical pimecrolimus cream. Majority showed good disease control with mild and transient side effects.	Topical pimecrolimus treatment led to good disease control in majority of erosive lichen planus cases.
Romero Ramírez et al.	2005	Case Report	Presented a case of childhood vulvar lichen planus.	Case report of vulvar lichen planus in a prepubertal girl.
Rotsztejn et al.	2007	Case Report	Reported a rare case of erosive lichen planus with large lesions in the vulvar area and vagina.	Rare localization of erosive lichen planus in vulvar region and vagina.
Hundley et al.	2011	Case Report	Described a case of vaginal agglutination mimicking lichen planus in a woman with chronic graft‐versus‐host disease.	Severe vaginal GVHD leading to agglutination, managed with multimodal therapy.
Lewis et al.	2013	Case Report	Explored histopathologic patterns secondary to the topical application of EMLA® on vulvar epithelium in three cases.	Vesicle and blister formation after EMLA application in patients with underlying erosive or burnt‐out lichen planus.
Yeo and Ormerod	2016	Case Report	Presented two cases of recalcitrant erosive lichen planus that improved with oral tacrolimus.	Refractory erosive lichen planus improved with oral tacrolimus after multiple failed treatments.
Day et al.	2017	Original Research	Identified 31 cases of comorbid vulvar lichen planus and lichen sclerosus. Central erythema and peripheral pallor were common clinical findings.	Comorbidity of vulvar lichen planus and lichen sclerosus with common clinical findings.
Poon et al.	2017	Case Report	Successfully treated erosive penile lichen planus with oral acitretin after failure of other therapies.	Severe, recalcitrant erosive penile lichen planus treated with oral acitretin.
Day et al.	2018	Original Research	Investigated the association between vulvovaginal lichen planus and squamous cell carcinoma.	Association between vulvovaginal lichen planus and squamous cell carcinoma.
Miotti et al.	2020	Case Report	Presented a case report of autologous micrografts and methotrexate in plantar erosive lichen planus.	Successful treatment of plantar erosive lichen planus with autologous micrografts and methotrexate.
Sadownik et al.	2020	Original Research	Explored the qualitative experiences of women living with chronic vulvar dermatoses.	Qualitative exploration of the impact of chronic vulvar dermatoses on women's lives.
Boch et al.	2021	Original Research	Conducted a retrospective analysis of clinical characteristics and patient‐reported outcomes in vulval lichen planus.	Clinical characteristics and outcomes in vulval lichen planus, with key morbidities identified.
Kanneganti et al.	2021	Case Report	Managed recurrent vaginal obliteration due to severe erosive lichen planus surgically.	Surgical management of recurrent vaginal obliteration in severe erosive lichen planus.
Lyra et al.	2021	Original Research	Examined erosive vulvar lichen planus and its risk of vulvar neoplasia in a cohort of 127 women.	Risk of vulvar neoplasia in women with erosive vulvar lichen planus.
Rezzag‐Mahcene et al.	2021	Case Report	Successfully treated recalcitrant genital lichen planus with secukinumab.	Successful treatment of recalcitrant genital lichen planus with secukinumab.
Skullerud et al.	2021	Original Research	Described the study protocol for the AP‐GELP trial, investigating apremilast for genital erosive lichen planus.	Protocol for a trial investigating apremilast in genital erosive lichen planus.
Vermeer et al.	2021	Original Research	Analyzed the use of hydroxychloroquine as a systemic treatment in erosive lichen planus of the vulva and vagina.	Use of hydroxychloroquine in systemic treatment for erosive lichen planus of the vulva and vagina.
Amsellem et al.	2022	Original Research	Retrospective study of 89 cases of male genital lichen planus, providing insights into clinical characteristics and treatment patterns.	Clinical characteristics, pathology results, complications, and treatment patterns of male genital lichen planus.
Chernova et al.	2022	Case Report	Presented a case record of complicated vulvovaginal‐gingival syndrome in a young patient.	Case record of a young patient with complicated vulvovaginal‐gingival syndrome.

Among case reports and series, EGLP resulted in symptoms like severe pain, pruritus, burning sensations, and sexual dysfunction. Examinations showed eroded, ulcerated, erythematous or white plaques on genital sites like the glans penis, labia, vaginal introitus, and perianal regions. Concurrent oral mucosal involvement was observed in 16–53% of EGLP cases. Associated autoimmune conditions were also documented.

Refractory EGLP resistant to multiple standard treatments was a major theme. Various systemic immunomodulators were trialed, including oral tacrolimus, secukinumab, acitretin, hydroxychloroquine, cyclosporine, mycophenolate mofetil, and methotrexate. More novel interventions included autologous micrografts, carbon dioxide laser ablation, and phytotherapy. Despite multiple attempted therapies, some cases showed continued progression.

Among original studies, first‐line treatment overwhelmingly involved high‐potency topical corticosteroids. However, up to 50% of genital lichen planus patients eventually needed additional systemic agents like retinoids, antimalarials, or immunosuppressants due to treatment failure with steroids alone. Surgical approaches were also employed for scarring complications like vaginal stenosis or phimosis, but symptoms frequently recurred postoperatively.

Long‐term outcomes included high rates of genital scarring (up to 62%), anatomical changes, and quality of life impairment even with treatment. Precancerous lesions or squamous cell carcinomas developed in 2–4% of patients.

One study found that patients’ perspectives centered around profound psychological suffering related to social isolation, stigma, and unresolved symptoms despite adhering to unpleasant topical treatments. Many described inadequate medical validation around their concerns.

In summary, current evidence shows EGLP causes substantial physical and psychosocial impacts not adequately treated with existing therapies. Further research is critically needed to better understand this challenging condition and identify more effective long‐term management strategies.

## 4 DISCUSSION

Table 3 summarizes the key details from each article, including the year of publication, study type, a brief summary of the article, and the main findings.

**Table 3:** Applied treatments and Interventions.

Article	Treatments and Interventions	Function
Vaskovskaya and Mirkhodzhaeva^13^	Information on specific treatments and interventions is not provided.	Investigated the occurrence of simultaneous involvement of buccal and genital mucous membranes in patients with lichen planus.
Edwards^14^	Topical steroids, antibiotics, and antifungals.	Management of vulvar lichen planus with topical steroids and antimicrobial agents.
Bermejo et al.^15^	Intralesional and topical steroids, systemic corticosteroids, antifungals.	Combination of intralesional, topical, and systemic steroids, along with antifungal agents.
Cribier et al.^16^	Systemic corticosteroids, antibiotics, and antifungals.	Treatment of peno‐gingival syndrome with systemic corticosteroids and antimicrobial agents.
Porter et al.^17^	Topical clobetasol and betamethasone, systemic prednisolone.	Treatment of erosive penile lichen planus with topical and systemic corticosteroids.
Mous et al.^18^	Topical and systemic corticosteroids, antibiotics, and antifungals.	Management of erosive genital lichen planus with corticosteroids, antibiotics, and antifungal agents.
Al‐Mutairi et al.^19^	Betamethasone oral mini‐pulse therapy.	Successful treatment of acute generalized lichen planus with oral mini‐pulse therapy.
Lonsdale‐Eccles and Velangi^20^	Topical pimecrolimus cream.	Successful disease control in erosive lichen planus with topical pimecrolimus.
Romero Ramírez et al.^21^	Topical steroids, antifungals.	Symptomatic relief with topical steroids and antifungal agents.
Rotsztejn et al.^22^	Topical and systemic corticosteroids.	Symptomatic relief with topical and systemic corticosteroids.
Hundley et al.^23^	Surgical restoration of patency, dilation, estrogen, clobetasol, and electocautery.	Multimodal therapy including surgical intervention, dilation, estrogen, and corticosteroid creams.
Lewis et al.^24^	Topical EMLA anesthetic, biopsy, corticosteroids.	Vesicle and blister formation after EMLA application. Confirmation of lichen planus despite changes.
Yeo and Ormerod^25^	Oral tacrolimus.	Improvement in refractory erosive lichen planus with oral tacrolimus.
Day et al.^26^	Topical steroids, compound clobetasol, surgical treatment, dilation, estrogen, clobetasol creams, electocautery.	Management of vulvar lichen planus and lichen sclerosus with topical and surgical interventions.
Poon et al.^27^	Topical steroids, oral prednisolone, oral acitretin, hydroxychloroquine, circumcision, and frenuloplasty.	Treatment with topical and systemic steroids, acitretin, and surgical intervention.
Day et al.^28^	Postoperative topical corticosteroids, referral for LS management.	Topical corticosteroids postoperatively. Possible referral for lichen sclerosus management.
Miotti et al.^29^	Topical steroids, antibiotics, immunosuppressants, surgical debridement, autologous micrografts, skin graft.	Multifaceted treatment including topical and systemic agents, surgical debridement, and skin grafting.
Sadownik et al.^30^	Information on specific treatments and interventions is not provided.	Qualitative exploration of women's experiences with vulvar dermatoses.
Boch et al.^31^	Topical corticosteroids, systemic therapies, surgery.	Treatment with topical corticosteroids, systemic immunomodulators, and surgical interventions.
Kanneganti et al.^32^	Surgical management, dilation, estrogen, and steroid creams.	Surgical intervention, dilation, and use of estrogen and steroid creams for recurrent vaginal obliteration.
Lyra et al.^33^	Ultrapotent topical corticosteroids, alternative treatments, surgery.	Topical and systemic corticosteroids, alternative treatments, and surgery for vulvar lichen planus.
Rezzag‐Mahcene et al.^34^	Secukinumab.	Successful treatment of genital lichen planus with secukinumab.
Skullerud et al.^35^	Topical corticosteroids, topical tacrolimus, oral acitretin, steroids.	Treatment with topical and systemic corticosteroids, tacrolimus, and oral acitretin.
Vermeer et al.^36^	Hydroxychloroquine, topical treatments.	Use of hydroxychloroquine as a systemic treatment for erosive lichen planus, with concomitant topical therapies.
Amsellem et al.^37^	Topical and oral corticosteroids, acitretin.	Topical and oral corticosteroids, acitretin, and surgical options for male genital lichen planus.
Chernova et al.^38^	Systemic corticosteroids, antibiotics, antifungals.	Treatment of complicated vulvovaginal‐gingival syndrome with systemic corticosteroids, antibiotics, and antifungal agents.

EGLP is a chronic, inflammatory dermatosis that poses significant challenges in terms of diagnosis and management. In this systematic review, we explored a diverse range of treatment modalities reported in 26 articles encompassing different aspects of EGLP. The following discussion provides a comprehensive overview and critical analysis of these therapeutic interventions.

### 4.1 Topical corticosteroids

Topical corticosteroids such as clobetasol and betamethasone remain the foundation of treatment for EGLP. Numerous studies over recent decades have demonstrated the efficacy of these agents in providing symptom relief and disease control.^13^, ^14^, ^15^, ^17^, ^18^, ^21^, ^22^, ^23^, ^26^, ^31^, ^36^ Both potent and very potent topical corticosteroids applied topically have proven capable of managing EGLP symptoms, as showcased in research by Porter et al.^17^ and Lonsdale‐Eccleset al.^20^ However, despite their undisputed utility as a cornerstone of treatment, concerns exist regarding adverse effects from prolonged use of topical corticosteroids. Such concerns have motivated investigations into therapeutic alternatives and corticosteroid‐sparing agents to use alongside or in place of topical steroids in EGLP management.

### 4.2 Systemic corticosteroids

In severe presentations of EGLP, systemic corticosteroids have been utilized as treatment.^14^, ^15^, ^21^, ^22^, ^26^ Both oral prednisolone and mini‐pulse therapy using betamethasone have demonstrated significant improvement in acute and generalized EGLP cases. A case study by Al‐Mutairi, Omran, and Mohammad presents a notable approach using betamethasone pulse therapy, showcasing systemic corticosteroids’ potential efficacy for severe EGLP.^19^ However, given the considerable risks associated with systemic steroid use, their administration requires judicious consideration on a case‐by‐case basis along with close monitoring for adverse effects. While systemic corticosteroids can effectively manage flares in some patients, their high side effect profile demands weighing benefits against risks before prescribing.

### 4.3 Alternative immunomodulatory agents

Several recent studies have explored alternative immunomodulatory medications for managing EGLP, including tacrolimus, pimecrolimus, secukinumab, and apremilast.^20^, ^25^, ^28^, ^34^ These emerging therapies demonstrated promise in steroid‐refractory EGLP cases, providing new options for treatment‐resistant disease. Both tacrolimus and pimecrolimus have been positioned as alternatives to corticosteroids in recalcitrant cases.^25^ Differences between the agents emerge upon comparison, with tacrolimus often preferred due to improved tolerability. The variety of choices indicates an increasing repertory of immunomodulators for customized EGLP treatment depending on patient conditions. While topical and systemic corticosteroids remain first‐line, these alternatives offer hope for patients unresponsive to or intolerant of steroid therapy.

### 4.4 Surgical interventions

In severe, anatomically complicated cases of EGLP, surgical interventions have been utilized as necessary components of disease management. Such procedures have included dilation, electrocautery, circumcision, and urethral advancement.^23^, ^27^, ^32^, ^33^, ^37^ Studies by Kanneganti et al. and Lyra et al. highlight the potential viability of surgery for refractory EGLP, particularly when combined with adjunctive therapies like anti‐adhesion gels and prolonged topical corticosteroids.^32^, ^33^ This demonstrates the value of a multifaceted approach, incorporating surgical division and postoperative pharmacological measures to promote healing in complicated cases unresponsive to more conservative treatment. While most EGLP can be managed medically, surgical interventions remain an option alongside systemic and topical immunomodulators in severe, refractory presentations with anatomical factors precluding healing.

### 4.5 Hydroxychloroquine

Recent research by Vermeer et al. explored the antimalarial medication hydroxychloroquine (HCQ) as a novel systemic therapy for erosive lichen planus vulvae (ELPV), a subtype of EGLP.^36^ Their study demonstrated HCQ's promising efficacy in ELPV, supporting consideration of its use for broader EGLP management as well. As an already approved therapy with a favorable safety profile, HCQ represents a potentially effective and well‐tolerated oral treatment option. While Vermeer et al.‘s research indicates HCQ may be a viable systemic alternative, further investigations into its therapeutic value specifically for EGLP are imperative to cement its role given the scarcity of current data. If effectiveness findings hold true in other cohorts, then new systemic medicines like HCQ might increase the EGLP armamentarium as traditional therapies have major side effects.

### 4.6 Patient experience and quality of life

EGLP substantially impacts patient quality of life, going beyond physical symptoms alone.^30^ Qualitative research exploring patient experiences reveals suffering, isolation, and disruption of daily functioning as common themes, underscoring the need for holistic care. As Boch et al. elucidate, appreciating EGLP's effects on overall well‐being is critical, as quality of life remains markedly impaired even in treated patients due to psychosocial factors compounding physical manifestations.^31^ These patient‐centered insights highlight substantial unmet needs, signaling an imperative for multifaceted approaches addressing both symptom control as well as supporting patients’ emotional, social, and functional capacities in the face of a devastating diagnosis. Comprehensive EGLP care must encompass more than medications alone.

### 4.7 Emerging breakthroughs and future directions

Recent advances showcase the rapidly evolving therapeutic landscape for EGLP. Targeted immunomodulators like secukinumab and apremilast have emerged as breakthrough options, although establishment of their long‐term safety and efficacy will require further research. Still, these drugs represent a change toward specialist therapy beyond traditional approaches. Preliminary findings introduced by Amsellem et al. and Skullerud et al. signal additional paradigm‐changing developments on the horizon.^35^, ^37^ Both anti‐IL‐17 antibodies and phosphodiesterase‐4 inhibitors represent exciting novel directions, underscoring the need to accelerate investigations into new medical treatments while outcomes remain suboptimal for many with EGLP. Though once a therapeutic desert, the field now overflows with promising early data on various experimental agents warranting illumination through rigorous studies.

### 4.8 Limitations and challenges

While the reviewed studies offer valuable preliminary insights, several limitations temper conclusions and reveal key challenges that remain. Heterogenous study designs, small sample sizes, and variability in outcome measures preclude effective comparison and validation of findings across EGLP clinical trials. As Day et al. emphasize, recognizing common comorbidities like lichen sclerosis also poses an ongoing obstacle.^28^ Furthermore, Lyra et al. highlight the lack of consistent management guidelines as a central concern needing addressed.^33^ Overall, the field continues to face hurdles like suboptimal methodological consistency, knowledge gaps regarding complication profiles, and lack of standardized treatment protocols. Moving forward requires multicenter collaborative efforts to execute large‐scale standardized trials that help elucidate optimal diagnostic and therapeutic algorithms for this heterogeneous, comorbidity‐laden disease. Though progress continues, it is incumbent upon researchers to reinforce foundations through methodologically rigorous studies while exploring evolving treatments for EGLP.

## 5 CONCLUSION

Effective management of EGLP requires a multifaceted and individualized approach, given the complexity and chronic nature of the condition. Our review underscores the importance of conventional topical corticosteroids, which remain a cornerstone in managing EGLP symptoms. However, the emergence of new therapeutic options, including immunomodulators and alternative systemic agents, offers promising avenues for patients unresponsive to standard treatments.

The significant physical and psychosocial impacts of EGLP, as revealed through patient experiences, highlight the need for comprehensive care strategies that address both symptom management and quality of life. The variability in treatment responses and the high rates of scarring and complications call for continued research into more effective and tailored treatment protocols.

Future studies should focus on large‐scale, standardized trials to better understand the efficacy and safety of emerging therapies and to develop optimized, evidence‐based management strategies for EGLP. Addressing the methodological limitations and exploring innovative treatment options will be crucial in advancing the care of patients with this challenging condition.

## AUTHOR CONTRIBUTIONS

**Borna Safari‐Kish:** Supervision; Conceptualization; Writing—original draft; Writing—review and editing; Data curation; Software; Visualization. **Matin Bidares:** Investigation; Validation; Visualization; Formal analysis; Project administration; Writing—original draft. **Shirin Zaresharifi:** Conceptualization; Methodology; Software; Data curation; Resources; Investigation; Visualization. **Hesam Malekzadeh‐Shoushtari:** Conceptualization; Validation; Methodology; Formal analysis; Software; Funding acquisition; Writing—original draft. **Mahsa Aziz:** Conceptualization; Project administration; Formal analysis; Software; Methodology; Validation; Investigation; Writing—original draft. **Mahsa Salehi:** Data curation; Conceptualization; Investigation; Writing—review and editing; Project administration; Formal analysis; Resources. **Khatere Zahedi:** Supervision; Conceptualization; Writing—original draft; Data curation; Software; Writing—review and editing; Visualization.

## CONFLICT OF INTEREST STATEMENT

The authors assert that they have no conflicting interests.

## ETHICS STATEMENT

None of the authors have conducted any research involving human subjects or animals that is detailed in this article.

## INFORMED CONSENT

There is no need for informed consent.

## TRANSPARENCY STATEMENT

The lead author Borna Safari‐Kish, Khatere Zahedi affirms that this manuscript is an honest, accurate, and transparent account of the study being reported; that no important aspects of the study have been omitted; and that any discrepancies from the study as planned (and, if relevant, registered) have been explained.

## Data Availability

The data that support the findings of this study are available from the corresponding author upon reasonable request. The data that support the findings of this study are available from the corresponding author upon reasonable request. All authors have read and approved the final version of the manuscript. Borna Safari‐Kish and Khatere Zahedi had full access to all of the data in this study and takes complete responsibility for the integrity of the data and the accuracy of the data analysis.
